# The Translation and Cross-Cultural Adaptation of the Pregnancy Physical Activity Questionnaire: Validity and Reliability of a Serbian Version (PPAQ-SRB)

**DOI:** 10.3390/healthcare10081482

**Published:** 2022-08-07

**Authors:** Marija Rovcanin, Svetlana Jankovic, Zeljko Mikovic, Sandra Sipetic Grujicic, Ivana Rudic Biljic Ersk, Milan Lackovic, Dejan Dimitrijevic, Sara Simanic, Isidora Vujcic

**Affiliations:** 1 Clinic for Gynecology and Obstetrics, Narodni Front, Kraljice Natalije 62, 11000 Belgrade, Serbia; 2 Faculty of Medicine, University of Belgrade, Dr Subotica Starijeg 8, 11000 Belgrade, Serbia; 3 Institute of Epidemiology, Faculty of Medicine, University of Belgrade, Visegradska 26, 11000 Belgrade, Serbia; 4 Clinical Hospital Center, Dr Dragiša Mišović, Heroja MilanaTepica 1, 11000 Belgrade, Serbia

**Keywords:** physical activity, pregnancy, Pregnancy Physical Activity Questionnaire, validity, reliability, Serbian population

## Abstract

Exercise during pregnancy has a positive effect on the health of both pregnant women and their fetuses. This study aimed to translate the Pregnancy Physical Activity Questionnaire (PPAQ) into the Serbian language and assess its validity and reliability among Serbian pregnant women. The study was conducted between October 2020 and March 2021 at the Obstetrics and Gynecology Clinic (Narodni Front), in Belgrade, Serbia. The PPAQ was translated according to a standardized methodology, and its internal consistency and construct and concurrent validity were assessed. The mean PPAQ score for the total amount of physical activity was 37.72 MET-h/week^−1^. Exploratory factor analysis of the Serbian PPAQ identified six factors similar to the original questionnaire that explained 70.26% of the data variance. The Cronbach’s alpha coefficient of the Serbian version of the PPAQ was 0.69. The two-week intraclass correlation coefficient (ICC) scores ranged from 0.768 to 0.930. We tested the evidence to assess the concurrent validity of the Serbian version of PPAQ (PPAQ-SRB) correlations with the International Physical Activity Questionnaire-long form (IPAQ-LF), and all domains of the PPAQ were significantly correlated with domains of the IPAQ-LF. The findings of our reliability and validity evaluation are consistent with those of prior studies, indicating that the PPAQ was successfully translated and implemented in the Serbian population and that its reliability was acceptable.

## 1. Introduction

Physical activity (PA) is an important preventive factor for noncommunicable diseases worldwide, and it contributes to the improvement of most health parameters; it reduces the risk of cardiovascular disease, diabetes mellitus, obesity, sarcopenia, osteoporosis, and cognitive disorders as well as certain malignancies [1,2]. The health benefits of PA are reflected in its contribution to primary and secondary preventive measures for chronic health conditions and premature mortality [3]. In this regard, PA is a particularly important prerequisite for the majority of the aspects of human health, especially the population in the reproductive period, and more specifically, pregnant women [4]. Pregnant women are particularly fragile and sensitive to external and internal influences, with a special focus on fetal health [5,6].

It is well known that exercise during pregnancy has a positive effect on the health of both the pregnant woman and the fetus [1,2]. Maternal benefits include reduced risk of excessive gestational weight gain, gestational diabetes mellitus, preeclampsia and hypertension, operative deliveries, postpartum and perinatal depression, and anxiety [7,8,9]. Additionally, PA improves maternal cardiorespiratory capacity and fitness level [9]. An excessive amount of weight gain during pregnancy has been found to have a significant correlation with a number of perinatal risk factors. These risk factors have the potential to affect not only the course of the pregnancy but also the outcome of the pregnancy, as well as the early motor development of the newborn [10].

Exercise during pregnancy is associated with a significantly higher incidence of vaginal delivery and a significantly lower incidence of cesarean deliveries [11]. Moreover, PA during pregnancy can improve women’s sense of mental well-being and quality of life and reduce fatigue levels during the postpartum period [12,13]. Other benefits of PA during pregnancy include lower rates of miscarriage, reduced length of labor, lower risk of injury and delivery complications, and prolonged postpartum recovery [7].

Women who are experiencing pregnancy-related disorders, most notably pregnancy-induced hypertension and gestational diabetes mellitus, may benefit from engaging in moderate-intensity activity in order to lower their blood pressure, improve the control of their gestational weight gain, improve their cells’ response to insulin, and improve their blood glucose levels [14]. Regular PA lowers blood glucose levels by increasing glucose uptake via insulin-independent pathways and lowers maternal insulin resistance [15]. Many other physical discomforts of pregnancy (e.g., lower back pain) might be managed and reduced in severity by engaging in PAs [16].

According to the annual report of Serbian population health research conducted in 2019 in our country, 45.6% of the female population were sitting/standing, 23.8% were excessively sedentary, 7.6% walked more than 30 min per day, and 7.2% performed aerobic exercise during the day [17]. PA among pregnant women in Serbia, one of the most susceptible demographic groups, has been insufficiently implemented through programs designed to achieve general health among this population. Additionally, in our country, not enough research has been conducted on the effects and benefits of exercise on pregnancy, nor has there been enough speculation about the most effective method of counseling pregnant women throughout the pregnancy course, which should primarily be provided by primary care physicians, to achieve the optimal levels of intensity and frequency in their exercise routines. According to a study performed during the first trimester of pregnancy among healthy women in our country, almost one-third of women had insufficient leisure-time PA, and one-quarter were insufficiently physically active during early pregnancy [18].

Official national recommendations on PA during pregnancy do not currently exist in Serbia, and therefore, we recommend and follow the global recommendations in our country. Guidelines for PA and exercise during pregnancy originated from different countries (UK, USA, Canada, and Australia) and regions (Asia-Pacific). The foremost consensus is that all women, with the exception of those with some medical or obstetric contraindications, should be physically active throughout pregnancy, with different views only on whether that should be initiated before or after the end of the first trimester [19,20,21,22,23,24].

The guidelines were made with a specific focus on the duration, frequency, intensity, and type of PA that is advised and which ones to avoid, along with methods for supervision with defined signs that call for the discontinuation of a certain activity. When prescribing exercises during pregnancy, the program should be highly individualized. A complete clinical evaluation should be performed to determine any potential health risks associated with exercise. With contraindications excluded, the PA status before pregnancy (sedentary or active) should be the basis for a decision on the initiation and intensity of PA. Previously inactive women are encouraged to begin gradually, at a lower intensity and duration, followed by a progressive increase in aerobic exercise from 15 to 30 min of exercise four times per week until it becomes a daily regimen. The upper limit for one session is determined by different methods depending on the guideline, most including the “talk test”, the Borg ratings of perceived exertion scale, and monitoring and keeping the heart rate within a certain value interval (around 60% of the maximum heart rate in most guidelines). Pregnant women who were regular exercisers before pregnancy and who regularly participated in a vigorous-intensity aerobic activity or high-intensity exercise programs can maintain their activities throughout pregnancy and the postpartum period. Additionally, it is necessary to discuss with healthcare practitioners how and when these activities should be adjusted over time. In general, these women do not need to substantially reduce their activity level; rather, they should modify their PA as pregnancy progresses and must be aware of proper hydration, the increased nutritional requirements of pregnancy and exercise, and the risk of heat stress through carrying out PA in a cool environment. Therefore, pregnant women should be physically active on most, preferably all, days of the week, with a minimum of 2–3 days per week. The duration should be at least 150 min of moderate PA, which should be achieved by being active on most days of the week for 30 min a day. Regarding the type of exercise, it is highly recommended to incorporate aerobic and resistance (strength) training activities. Aerobic exercises include walking, stationary cycling, swimming, and water aerobics, whereas resistance training includes exercises with weights and resistance bands. Although there is less evidence, yoga, gentle stretching, balance and posture practice, and pelvic floor muscle training are general recommendations, but they should be performed with caution and supervision. In addition, gradual warm-ups and cool-downs are always advised as steps that reduce the risk of injury by encouraging muscle support. Maintaining a decent level of fitness during pregnancy is a reasonable objective for aerobic conditioning during pregnancy, rather than striving for peak fitness. Some exercises should be avoided as they present a certain risk for the woman and/or the fetus, such as those involving physical contact (contact sports), danger of falling with rapid changes in direction and bouncing (cycling), or changes in pressure (such as sky diving or scuba diving). Maintaining adequate caloric and water intake before, during, and after exercise should not be overlooked when prescribing an exercise regime [19,20,21,22,23,24]. 

In 2004, Chasan-Taber et al. developed and validated the Pregnancy Physical Activity Questionnaire (PPAQ), which is a comprehensive and reliable self-administered questionnaire to determine the degree of PA during pregnancy [25]. This questionnaire was designed as a universal tool for measuring the amount of PA in pregnant women. The PPAQ is readily understood by responders who participate in a range of activities, making it valuable for epidemiological research and pregnancy health counseling. It has been translated into numerous languages and has been used in a variety of clinical trials. 

However, there is no Serbian version of the PPAQ, despite the fact that it is necessary for assisting researchers in monitoring and evaluating PA in our country, assessing correlations between PA during pregnancy and pregnancy outcomes in Serbia, and comparing data collected from pregnant women in Serbia with data collected from other countries. This would also assist in the establishment of prenatal PA recommendations to promote maternal and child health in Serbia, which is an imperative requirement.

## 2. Materials and Methods

### 2.1. Study Design 

This study was carried out with the purpose of proving the reliability and validity of a Serbian translation of the PPAQ, put forward by Chasan-Taber et al. [25]. The study approach was composed of two parts: a transcultural translation of the PPAQ from English to Serbian and an analysis of the reliability and validity of the Serbian version of the Pregnancy Physical Activity Questionnaire (PPAQ-SRB).

### 2.2. Participants

All consecutive healthy pregnant women who came for routine gynecological check-ups between October 2020 and March 2021 at the Obstetrics and Gynecology Clinic, Narodni Front, in Belgrade, Serbia, were recruited for the study. Gestational age was determined based on menstrual history or obstetrical findings. Women with a normal course of singleton pregnancy aged 18–40 years who spoke Serbian fluently were included in the study. The study excluded pregnant women with multiple pregnancies; a previous history of premature birth; any chronic diseases; any gestational complications such as oligohydramnios or placenta previa; any kind of systemic and/or local infection that could prevent the participant from being physically active across the timeframe that encompasses the period during which PA was assessed by the questionnaire; gestational diabetes mellitus; pregnancy-induced hypertension/preeclampsia; previous history of malignant, systemic, autoimmune, or musculoskeletal disorders, liver or renal disease, or restrictive lung disease; and verified psychiatric disorders. 

Moreover, women who denied participation (5), offered less than 90% of the required replies (7), or did not meet the inclusion criteria (8) were not included in the study. Thus, the data from 20 participants were excluded from the study. Finally, a sample of 150 women who were eligible to participate in the study were included.

The minimum sample size to be included in this study was calculated following the recommendation of a minimum subject-to-item ratio of at least 5:1 in exploratory factor analysis (EFA) [26]. Since the final version of the PPAQ-SRB comprises of 30 items available for factor analysis, the study sample consisted of 150 women.

### 2.3. Ethical Consideration

The study was implemented in accordance with the International Code of Medical Ethics of the World Medical Association (Declaration of Helsinki), and written informed consent was obtained from the mothers after the nature and objectives of the study were fully understood by them. This study was approved by the ethical committee of the clinic (decision number: 05006-2021-1525; date of approval: 17 September 2020).

### 2.4. General and Clinical Data Collection

The general questionnaire gathered information on the respondents’ sociodemographic characteristics (age, level of education, occupation, and socioeconomic status), clinical characteristics (information on previous and current pregnancy), and lifestyle (smoking, alcohol, and drug abuse).

### 2.5. Questionnaire Data Collection

The first stage of our study involved the translation of the PPAQ from English to Serbian as well as cultural adjustments. Validity and reliability assessments were performed in the second section, and concurrent validity with the International Physical Activity Questionnaire–long form (IPAQ-LF) was measured in the third section.

Originally, the PPAQ was a semiquantitative questionnaire containing 36 questions. The first three questions enquired about the date of completion of the questionnaire, the date of the last menstrual period, and the expected date of delivery, followed by 33 questions covering different types of PA, including household tasks/caregiving (13 items), work/occupational (5 items), sports/exercise (9 items, including 2 open-ended questions), transportation (3 items), and physical inactivity/rest (3 items). 

Participants were asked to select the category that best approximated the amount of time spent on 33 activities during the current trimester. Durations ranged between 0 and ≥6 h per day and 0 and ≥3 h per week. Two additional questions were open for participants to add other types of PA that were not included in the questionnaire. The duration of time spent on each activity was multiplied by its intensity to measure the average weekly energy expenditure (metabolic equivalent MET-h∙week^−1^) attributable to each activity, according to the PPAQ instructions given by Taber et al. [25].

For questions 4–11, 14–16, and 20–22, the duration scores corresponded to the following duration categories, 0, 0.12, 0.50, 1.0, 2.0, and 3.0, and were multiplied by 7 to be converted into weekly data. For questions 12–13 and 32–36, the duration scores corresponded to the duration categories 0, 0.12, 0.50, 2.0, 4.0, and 6.0 and were also multiplied by 7 to be converted into weekly data. For questions 17–19 and 23–31, the duration scores corresponded to the following duration categories: 0, 0.12, 0.50, 1.0, 2.0, and 3.0, and these values were already in weekly form. The intensity of the average weekly energy consumption was calculated by multiplying the specific MET assigned to each question according to PPAQ instructions. The total activity was computed as the summation of the durations and intensities of all activities.

The questionnaire classifies activity by intensity: sedentary (sum of (duration × intensity) for questions 12–13 and 30–31 if open-ended activities were <1.5 METs); light (sum of (duration × intensity) for questions 4, 5, 7, 11, 15–18, 20, 22, 32, and 34 and questions 30 and 31 if open-ended activities are 1.5–<3.0 METs); moderate (sum of (duration × intensity) for questions 6, 8–10, 14, 19, 21, 23, 24, 27–29, 33, 35, and 36 and questions 30 and 31 if open-ended activities are >3.0–<6.0 METs); or vigorous (sum of (duration × intensity) for questions 25 and 26 and questions 30 and 31 if open-ended activities are >6.0 METs). 

According to the PPAQ, each activity was classified by type: household/caregiving (sum of (duration × intensity) for questions 4–10, 14–19); occupational (sum of (duration × intensity) for questions 32–36); sports/exercise (sum of (duration × intensity) for questions 23–31), transportation (sum of (duration × intensity) for questions 20–22); and inactivity (sum of (duration × intensity) for questions 11–13).

Permission for the translation and application of the PPAQ was received from the author, and an internationally accepted approach for the cross-cultural adaptation of questionnaires through the five stages (forward translation, synthesis, back-translation, expert committee review, and pretesting) recommended by Guillemin et al. was used [27]. Initially, independent translations from English to Serbian (the forward translations) were performed by two translators, one who worked in the medical field and the other who did not, and they were both unaware of the objectives of this study. A third translator was charged with reconciling the translations. The translation was conceptual rather than literal, with the objective of using the common language used by the target group to minimize professional jargon, lengthy phrases, and any words that participants would find objectionable. A fourth translator who did not work in the health sector and was blinded to the original questionnaire completed a backward translation (from Serbian to English). All translators were Serbian native speakers and fluent in both English and Serbian. Finally, the fifth version of the PPAQ in Serbian was established by a language coordinator and clinical expert team. 

The English measure (gallons) is not commonly used in our population; therefore, it was replaced with liters. All participants were in the third trimester of the pregnancy. The two open-ended questions (30 and 31) remained unanswered in our study; therefore, they were not included in the final analysis. Furthermore, one item (18 ″Mowing lawn while riding”) was discarded because of cultural differences since this type of mover is not commonly used in Serbia in the general population, and among pregnant women especially. 

The final draft was accepted after inconsistencies and conflicts were corrected and potential differences and alternatives were reviewed. Subsequently, the completed version was compared with the original English text, and the required improvements were made. To limit bias and subjective contributions, the document’s objectives and goals were not disclosed to the translators.

To check the understanding and interpretation of the translated items by the Serbian population, the questionnaire was administered to 15 pregnant women. As there were no remarks on the clarity or understanding of the items, the final version was generated and applied in this study. The final version of the PPAQ-SRB comprises 30 activities, and the self-administration of the PPAQ-SRB takes approximately 5–15 min.

### 2.6. Data Analysis

Descriptive statistical methods were used to characterize the patients’ sociodemographic and clinical data. Continuous variables are presented as averages (±standard deviation), and percentages are used for categorical variables. To describe the PPAQ-SRB scale, we analyzed the minimum and maximum scores for all PPAQ-SRB subscales and each item. All statistical analyses were performed using the IBM SPSS Statistics for Windows, version 23.0 (IBM Corp., Armonk, NY, USA). All 150 participants were included in this analysis, except for the ICC calculation, for which only 30 participants were included.

To assess construct validity, principal component analysis with varimax rotation (EFA) was performed. A factor was considered significant if its eigenvalue was >1.0. Furthermore, the items were retained based on a factor loading of >0.4 [28].

The reliability of the PPAQ-SRB was measured based on its internal consistency using Cronbach’s alpha coefficient and the intraclass correlation coefficient (ICC). The Cronbach’s alpha coefficient was evaluated for each factor. Values above 0.7 were recognized as statistically adequate. ICC was calculated by comparing scores in the second stage, within a 2-week interval after the researchers administered the questionnaire to 30 pregnant women participating in the study. ICC less than 0.5 was considered poor, between 0.5 and 0.75 was considered moderate, between 0.75 and 0.9 was considered good, and greater than 0.90 was considered excellent [29].

Concurrent validity was assessed by correlating PPAQ-SRB scores with the self-reported IPAQ-LF scores using Spearman’s correlation coefficients. The IPAQ-LF was developed by an international consensus group in Geneva in 1998–1999 [30] followed by extensive reliability and validity testing, which confirmed its validity across over 10 countries on both the long and short forms.

The reliability of the Serbian version of the IPAQ-LF questionnaire was confirmed in the study by Milanovic et al. conducted among older adults. ICCs in the Serbian version of the questionnaire ranged from 0.53 to 0.91, and we used it in this research [31]. The IPAQ-LF was also validated in the population of Serbian adolescents [32].

The IPAQ-LF consists of 27 questions for use in young and middle-aged adults, from 15 to 69 years old, and estimates an individual’s level of PA in the last 7 days. It also contains questions about sedentary habits. A metabolic equivalent task (MET) value was calculated for each domain and the total weekly PA level (MET-minutes/week) was calculated by summing MET values for each item. The following coefficients were used: 8.0 MET for vigorous PA; 4.0 MET for moderate PA; and 3.3 MET for walking PA. According to the IPAQ scoring instructions, four domains were calculated in MET-minutes/week as follows: work-related, the sum of walking, moderate, and vigorous scores at work; transport, the sum of walking and cycling scores for transportation; housework/gardening, the sum of the vigorous yard, moderate yard, and moderate inside chores scores; and leisure time, the sum of walking, moderate, and vigorous scores in leisure [33].

Vigorous activities were defined as activities in which participants breathe much harder than normal, moderate activities were those in which participants breathe somewhat harder than normal, and walking was not considered moderate PA. To be registered, an activity should last a minimum of 10 consecutive minutes.

Intensities for each item were calculated separately (vigorous activity, moderate activity, and walking) [33], and total scores for all walking, moderate, and vigorous PA were tallied as total weekly PA.

## 3. Results

### 3.1. Demographic Characteristics of the Analyzed Sample

Table 1 presents the basic descriptive parameters of the participants. The enrolled group of participants was homogeneous in terms of maternal age and duration of pregnancy. Almost half of the participants were in their first pregnancy (n = 98), and more than a third of the participants (n = 52) had had at least one pregnancy in the past. None of the participants in this study underwent in vitro fertilization. Most of the participants had a middle or high level of education. The majority of participants were from urban areas. A total of 75.3% of participants were married, 92% were employed, and 67.3% rated their financial situation as good. Almost one third of the participants were cigarette smokers, and none of the participants were alcohol or drug abusers during pregnancy.

### 3.2. PPAQ-SRB Scores for Amount of PA

The mean values for total activity and its two subscales, intensity and type, on the PPAQ-SRB are shown in Table 2. The study results indicated that the mean PPAQ-SRB score for the total amount of PA was 37.72 MET-h/week^−1^ and the mean score for total activity (light-intensity activity or above) was 33.71 MET-h/week^−1^. By PA intensity, the largest median values were achieved for light-intensity (15.60 MET-h/week^−1^) and moderate-intensity (17.85 MET-h/week^−1^) activity, while the lowest median was obtained for vigorous-intensity activity (0.26 MET-h/week^−1^). In terms of the type of PA, participants expended the most energy during occupational” and household/caregiving activities (13.79 MET-h/week^−1^ and 13.10 MET-h/week^−1^, respectively), whereas sports/exercise received the lowest energy expenditure (1.02 MET-h/week^−1^).

### 3.3. Construct Validity of PPAQ-SRB

In the EFA of the Serbian PPAQ, which included 30 items, we identified 6 factors similar to the original questionnaire by type of activity (Table 3). The extracted factors explained 70.26% of the variance in data. The Kaiser–Mayer–Olkin (KMO) statistic was 0.821, indicating an adequate sample size for performing EFA, and the result of Bartlett’s test of sphericity was *p* < 0.001. The first factor in our analysis consisted of seven items, the second of six, the third and fourth factors of five each, the fifth of four, and the sixth of three items. The commonality indices of the included items ranged from 0.518 to 0.802, indicating adequate communality. Factor 6 corresponds to the original factor inactivity and factor 4 with occupational activity. Together, factors 1 and 3 correspond to the original factor household/caregiving activity, indicating that Factor 1 is related to caregiving activity and Factor 3 to household activity. Factor 2 included items related to transportation and walking activities for fun or exercise. Factor 5 included sports/exercise but not walking, as was the case in the original questionnaire.

### 3.4. The Reliability of the PPAQ-SRB

The Cronbach’s alpha coefficient of the PPAQ-SRB was 0.69, which is slightly below the acceptable cut-off of 0.7. Cronbach’s alpha for all PPAQ-SRB subscales was adequate (all above 0.75). The two-week ICC scores ranged from 0.768 to 0.930, with the lowest value recorded for household activity and the highest for caregiving activity (Table 4).

### 3.5. Concurrent Validity of the PPAQ-SRB

Correlation coefficients between the domains of PPAQ-SRB and of IPAQ-LF are presented in Table 5, and there were statistically significant correlations in all the domains. Overall, we observed mainly moderate and significant correlations between the total score and total activity (light or above) on the PPAQ-SRB and IPAQ-LF domains (Spearman’s rho ranged from 0.345 to 0.625). The only low correlations were found between the summary measures of PPAQ-SRB and the total vigorous and sedentary activity on the IPAQ. A moderate to high and significant correlation was also found between vigorous activity on the PPAQ-SRB and transport (Spearman’s rho 0.699), leisure activity (Spearman’s rho 0.638), and total moderate activity (Spearman’s rho 0.620) on the IPAQ-LF. The sport/exercise and transport domains on the PPAQ-SRB also showed mainly high and significant correlations with transport, leisure activity, and total moderate activity on the IPAQ-LF. Sedentary activity (Spearman’s rho 0.862) and inactivity (Spearman’s rho 0.896) on the PPAQ-SRB showed significant and strong correlations with the sedentary activity domain on the IPAQ-LF.

## 4. Discussion

Our study indicates that the PPAQ translated into Serbian has good psychometric properties among healthy pregnant women in Serbia. Furthermore, the PPAQ-SRB has a six-factor structure that is very similar to the original questionnaire by type of activity. The questionnaire also demonstrated adequate internal consistency.

The PPAQ-SRB has acceptable characteristics, and we found that the six-factor solution was suitable for our study because of the adequate sample size and proven suitability of our data, achieved utilizing KMO statistics, which were larger and comparable with the construct validity of the Persian version of the PPAQ [34]. Therefore, the PPAQ-SRB could be used to assess the level of PA among pregnant women in Serbia, which was supported by the fact that women clearly comprehend and respond to PPAQ-SRB.

Cronbach’s α coefficient for the whole scale was 0.69, slightly below the acceptable cut-off of 0.7. Similarly, a Danish study reported a Cronbach’s alpha for the PPAQ of 0.70 [35]. In order to raise the Cronbach’s alpha coefficient, we calculated the value it would be when individual items were deleted from the scale (Table S1). Removal of any item except item 35, “Walking quickly at work while carrying things”, resulted in a lower Cronbach’s alpha, indicating that we can remove only this one item in order to achieve an acceptable overall Cronbach’s alpha above the established threshold of 0.70. Although deleting item 35 would increase Cronbach’s alpha to 0.703, we think that it would not improve the theoretical structure of the scale. In addition, the results of EFA indicated that item 35 should not be discarded due to adequate loadings on the relevant factor. All PPAQ subscales in our study also had Cronbach’s alpha coefficients above the standard cut-off of 0.7, and the highest coefficient was obtained for caregiving activities (0.919). However, it might be difficult to achieve a high overall Cronbach’s alpha for this scale because different types of physical activities were tested within one instrument. Taking into account all of the above, and that this questionnaire was validated and has been used for 18 years we decided not to remove question number 35 from the analysis. In our opinion, the Cronbach’s alpha coefficient for the whole scale demonstrated the acceptable internal consistency of the PPAQ in the Serbian population. In addition, Gardner indicated that the scale may consist of several clusters of items each estimating a distinct factor, and it will reveal internal consistency as long as every item correlates well with some other items [36,37]. He also pointed out that the scale needs to be unidimensional to provide an interpretable result and that it is important that all the items reflect the same construct when using a scale where a total score was obtained by summing the responses across items [36,37]. The Cronbach’s α is contingent upon the number of items comprising the subscales [38]. The value may be difficult to comprehend because it is dependent on the number of participants, variety of their responses, and sample size. Therefore, a larger sample than the current one would result in increased variability in estimated values and a higher total Cronbach’s α [39].

Furthermore, according to the Koo and Mae guidelines [29], the current study had strong test–retest reliability, with ICCs ranging from 0.768 to 0.930. This result is comparable with previous translations and adaptations of the PPAQ, such as those by Danes, Persians, and Vietnamese, as well as the original version [25,34,35,40]. Based on the activity categories, the reliability results were good for all types of activities except caregiving, which was excellent.

The ICC for occupational activity in our study showed good reliability, as it did in the studies of Chinese, Spanish, Vietnamese, Persians, and Poles [34,40,41,42,43]. In addition, the ICCs for household activity and sports/exercise activity showed good reliability, which was in line with other studies [34,35,40,42].

The assessment of PA type indicated that the lowest values were associated with household, transportation, and walking activities, emphasizing that these subscales compriseactivities that change rapidly during pregnancy, resulting in lower ICCs in a test–retest. In addition, the values for transportation and walking may demonstrate the distinctiveness of these types of activities and may be explained by the fact that pregnant women have planned doctor visits that may alter their daily behavior.

In this study, the highest ICC for a subscale was obtained for the caregiving subscale, which was expected given that this subscale covers actions that may be constant across time despite pregnancy. Moreover, this activity can be classified as routine. Based on our current study, we consider the reliability of the PPAQ-SRB to be acceptable.

Evidence for the concurrent validity of the PPAQ-SRB was provided by determining its relationship with the IPAG-LF by assessing the Spearman’s rank correlation, and almost all domains of the PPAQ significantly correlated with domains of the IPAQ-LF with moderate or high intensity.

The overall activity scores of our pregnant women were higher than overall scores observed in other studies [25,44,45,46]. In general, through criticism and advice, cultural norms and expectations often shape what pregnant women do with their bodies. In addition, certain stages of pregnancy are associated with unique barriers to being physically active. Many women feel more confident in terms of health at the end of the first and beginning of the second trimesters, which results in an increase in their PA. In the early stages of pregnancy, the mother’s main concern was the developing fetus. However, in the later stages of pregnancy, women try to safeguard themselves by recognizing the many benefits of PA and anticipating physical and mental well-being during pregnancy, delivery, and the postpartum period [47]. In addition, the higher total activity in our country may be attributable to the higher education level of our study group and the greater awareness of the importance of maintaining a healthy lifestyle during pregnancy. The average age of our study group could be another reason for the higher total activity score than that of studies compared. 

Scores for light- and moderate-intensity activity in our study were found to be higher than in the U.S., while the median for vigorous-intensity activity was in accordance with the findings of prior studies in the United States, Japan, Vietnam, China, Saudi Arabia, and South Korea [25,40,41,44,45,46]. Women’s modifying the intensity and type of their activities in pregnancy, especially through the later stages of pregnancy, might be reflected in their lower scores for vigorous activities and sports and recreational activities.

In our study group, almost two thirds of the women were in their first pregnancy and living in a community with their partner and did not work during pregnancy, so household/caregiving activities was the major PA during the day in addition to recreational walking and rest. 

The median for sports/exercise was consistent with the results from other studies, which showed that the amount of energy expended during sports and exercise by pregnant women is relatively modest [25,35,40,41,42,44,45,48]. This could be because the participants in our study group were in the last trimester of pregnancy and were already preparing for childbirth through activities that consumed less energy and through continued daily activities. In addition, the lower scores for sports/exercise during pregnancy may be attributed to preconceived notions about a woman’s diminished mobility during pregnancy as well as a concern about potential accidents sustained when participating in this form of PA [49].

Sedentary behavior was almost three times less prevalent in our study than in the U.S., reflecting that almost two thirds of our participants were in their first pregnancy and that in our country, there is a firm traditional belief that pregnant women should walk recreationally every day, eat healthily, and manage excessive weight gain during pregnancy. Sedentary activity during pregnancy is associated with many potential risk factors for the outcome of the pregnancy itself, and one of them is obesity.

Due to the homogeneity of the study sample, there are certain limitations to the current study that should be mentioned. The majority of participants in our research had a high school diploma or higher, which conforms to the common experience with questionnaire surveys. When a questionnaire is used to assess PA, the patient’s age, education level, occupation, and socioeconomic position strongly influence how the questions are understood and answered. It is important to emphasize that the sample is not necessarily representative of the entire population of pregnant women in our country, as there were very few participants with low socioeconomic and education levels.

In addition, the PPAQ-SRB is a self-reported assessment of PA, which may have been constrained by recall bias. The exclusion of high-risk pregnant women and the enrollment of only pregnant women who met the eligibility criteria and who voluntarily participated was a limitation for the representation of the general population of pregnant women. Furthermore, this research does not reflect all three trimesters uniformly, as our participants were only in the third trimester. If all three trimesters were equally represented, the findings might have been classified by trimester during the data analysis, thereby enhancing the research. Moreover, as previously mentioned, in our country, we strongly recommend and follow global recommendations in their absence for Serbia specifically. In addition, because the questionnaire may be used to determine the relative rank of activities in population-based studies, we believe that exclusion has no effect on the reliability or validity of this instrument in epidemiological research.

The advantages of this work include a systematic and comprehensive procedure for transcultural adaptation and tightly standardized techniques for PPAQ validation. Moreover, the compliance of all participants and the ease of completing this newly adapted tool, excluding the validation process from the very beginning of all questionnaires with missing data, were additional advantages of this study.

For future studies that require a comprehensive evaluation of PA, a questionnaire and a pedometer or an accelerometer should be considered together and with high significance to acquire a broader view of the validity and reliability of the PPAQ-SRB and to add a more unbiased analysis approach. Therefore, in future research, we will compare the accuracy of our tool with that of an accelerometer or pedometer in a group of pregnant women, including those with high-risk pregnancies, to determine the most accurate self-reported tool for assessing the level of PA during pregnancy. Additionally, we believe that future studies with this instrument will offer early information to healthcare professionals about the present levels of PA in pregnant Serbian women and assist us in quantifying the levels of PA that avert depression symptomatology during and post-pregnancy among Serbian women and might help with investigating less common activities during pregnancy, including vigorous activities, which are rarely discussed.

## 5. Conclusions

Despite the previously stated limitations, it is possible to infer based on the findings of this study that the results of our reliability and validity evaluation are consistent with those of prior studies, indicating that the PPAQ was successfully translated and adapted in the Serbian population and that its reliability is acceptable. Moreover, information obtained from this questionnaire may be essential for the development and promotion of PA programs for pregnant women in order to attempt to prevent pregnancy complications by issuing specific guidelines regarding the importance and advantages of an active lifestyle during pregnancy and monitoring health attitudes and habits. In particular, the PPAQ-SRB would be valuable for future public policies aimed at addressing the health concerns of mother–offspring pairs, adjusting health promotion initiatives, and comparing PA across the world to conduct multinational collaborative studies in this field. Therefore, we recommend using the Serbian PPAQ to measure PA among pregnant Serbian women.

## Figures and Tables

**Table 1:** Descriptive data of the basic parameters for the pregnant women enrolled in the study.

Parameter	N = 150
	(x ± SD)
Maternal age (years)	29.5 ± 4.65
Gestational age (weeks)	39.4 ± 0.844
	Numbers (%)
Number of previous pregnancies:	
none	98(65.3)
≥1	52(34.7)
In vitro fertilization	0
Degree of education:	
Elementary school	2 (1.3)
High school	64 (42.7)
Undergraduate education	64 (42.7)
Postgraduate education	20 (13.3)
Capital residence:	137 (91.3)
Marital status:	
Married	113 (75.3)
Extramarital union	33 (22.0)
Divorced	1 (0.7)
Widow	0
Single	3 (2)
Employed:	
Yes	138 (92.0)
No	12 (8.0)
Self-rated financial status:	
Poor	2 (1.3)
Average	47 (31.3)
Good	101 (67.3)
Lifestyle:	
Cigarette smoker	45 (30.0)
Alcohol consumer	0
Drug addiction history	0

**Table 2:** Average scores on the Serbian version of PPAQ ^1^ according to domains in MET ^2^.

Variables(Unit: MET-h/week^−1^)	Min	Max	Mean	SD	Skewness	Kurtosis
The total activity score of PPAQ	9.52	82.80	37.72	14.67	0.40	−0.18
Total activity (light and above)	7.22	75.52	33.71	14.57	0.42	−0.30
**By intensity**						
Sedentary activity	0.00	8.82	4.01	2.74	0.27	−1.22
Light-intensity	3.54	39.70	15.60	6.85	0.59	0.37
Moderate-intensity	0.96	50.91	17.85	10.87	0.55	−0.19
Vigorous-intensity	0.00	2.70	0.26	0.45	3.12	11.37
**By type**						
Household/Caregiving	0.00	41.13	13.10	10.11	0.85	−0.22
Occupational	0.77	30.56	13.79	7.68	0.43	−0.40
Sports/exercise	0.00	6.41	1.02	1.36	2.29	5.33
Transportation	0.67	14.18	4.63	2.97	1.22	0.82
Inactivity	0.00	12.60	5.18	3.67	0.40	−1.06

^1^ PPAQ = Pregnancy Physical Activity Questionnaire; ^2^ MET = metabolic equivalent task.

**Table 3:** The rotated matrix of factor analysis with commonalities for each factor.

Factor Rotation ^1^ Matrix ^2^	1	2	3	4	5	6	Commonalities
Item 4	0.841						0.731
Item 5	0.808						0.658
Item 6	0.861						0.752
Item 7	0.815						0.699
Item 8	0.872						0.774
Item 9	0.810						0.677
Item 10	0.810						0.667
Item 11						0.803	0.673
Item 12						0.835	0.716
Item 13						0.836	0.721
Item 14			0.890				0.802
Item 15			0.800				0.663
Item 16			0.803				0.676
Item 17			0.824				0.698
Item 19			0.812				0.683
Item 20		0.823					0.788
Item 21		0.859					0.769
Item 22		−0.704					0.518
Item 23		0.795					0.757
Item 24		0.808					0.724
Item 25		0.799					0.657
Item 26					0.815		0.748
Item 27					0.819		0.797
Item 28					0.824		0.758
Item 29					0.802		0.679
Item 32				0.823			0.705
Item 33				−0.716			0.557
Item 34				0.862			0.754
Item 35				−0.700			0.589
Item 36				0.794			0.692

Extraction method = Principal component analysis; ^1^ Rotation method = Varimax rotation; ^2^ Six-factor model.

**Table 4:** Intraclass correlation coefficients and internal consistency of PPAQ-SRB ^1^ measured by Cronbach’s α coefficient.

Factor	Number of Questions	Cronbach α N = 150	ICC Coefficient ^2^ N = 30	95%CI
Factor 1: Caregiving activity	7	0.919	0.930	0.852–0.967
Factor 2: Transport and walking activity	6	0.757	0.773	0.523–0.892
Factor 3: Household activity	5	0.752	0.768	0.512–0.890
Factor 4: Occupational activity	5	0.815	0.815	0.611–0.912
Factor 5: Sports/exercise activity	4	0.867	0.841	0.665–0.924
Factor 6: Inactivity	3	0.784	0.798	0.575–0.904

^1^ PPAQ-SRB = Pregnancy Physical Activity Questionnaire-Serbian version; ^2^ ICC = Interclass correlation coefficient (95% confidence interval).

**Table 5:** Concurrent validity assessed by correlating oPPAQ-SRB ^1^ scores with IPAQ-LF ^2^ scores using Spearman’s correlation coefficient.

	IPAQ-LF (MET-min/week^−1^)								
	Work	Transport	Housework/Gardening	Leisure Activity	Total Walking	Total Moderate	Total Vigorous	Sedentary	Total PA
Total score of PPAQ (MET ^3^-min/week^−1^)	0.345 **	0.359 **	0.408 **	0.406 **	0.624 **	0.536 **	0.161 *	0.096	0.540 **
Total activity (light and above)	0.347 **	0.357 **	0.416 **	0.413 **	0.625 **	0.540 **	0.176 *	−0.054	0.553 **
**By intensity**									
Sedentary	0.018	0.012	−0.010	−0.032	0.006	0.002	−0.049	0.862 **	−0.044
Light	0.088	0.167 *	0.332 **	0.294 **	0.508 **	0.357 **	−0.088	0.028	0.278 **
Moderate	0.426 **	0.371 **	0.346 **	0.367 **	0.510 **	0.499 **	0.291 **	−0.064	0.567 **
Vigorous	0.064	0.699 **	0.069	0.638 **	0.245 **	0.620 **	0.270 **	0.007	0.528 **
**By type**									
Household/Caregiving	0.058	0.114	0.358 **	0.306 **	0.459 **	0.299 **	0.013	−0.171	0.275 **
Occupational	0.502 **	0.131	0.291 **	0.138	0.514 **	0.291 **	0.185 *	0.005	0.409 **
Sports/exercise	0.068	0.727 **	0.058	0.730 **	0.289 **	0.691 **	0.215 **	0.026	0.565 **
Transportation	0.156	0.771 **	0.087	0.558 **	0.321 **	0.650 **	0.216 **	0.059	0.581 **
Inactivity	0.062	0.036	−0.001	−0.027	0.004	0.029	−0.019	0.896 **	−0.022

^1^ PPAQ = Pregnancy Physical Activity Questionnaire; ^2^ IPAQ-LF = International Physical Activity Questionnaire-Long Form; ^3^ MET = metabolic equivalent task; * Statistical significance: *p* < 0.05; ** Statistical significance: *p* < 0.01.

## Data Availability

The data that support the findings of this study are available from the corresponding author (I.V.) upon reasonable request.

## Supplementary Materials

The following supporting information can be downloaded at: https://www.mdpi.com/article/10.3390/healthcare10081482/s1, Table S1: Cronbach’s α if item deleted (PPAQ-SRB).
