# Supplementary material for: The Translation and Cross-Cultural Adaptation of the Pregnancy Physical Activity Questionnaire: Validity and Reliability of a Serbian Version (PPAQ-SRB)
Source: Healthcare (Basel). 2022 Aug 7;10(8):1482. doi: 10.3390/healthcare10081482 (PMC9408768; doi:10.3390/healthcare10081482)
Supplement: Supplementary file 1 [file healthcare-10-01482-s001.zip › healthcare-1789600-supplementary.pdf]

Table S1. Cronbach's α if item deleted (PPAQ-SRB)

	Cronbach's α if item deleted
Item 4: Preparing meals (cook, set table, wash dishes)	,677
Item 5: Dressing, bathing, feeding children while you are sitting	,680
Item 6: Dressing, bathing, feeding children while you are standing	,675
Item 7: Playing with children while you are sitting or standing	,677
Item 8: Playing with children while you are walking or running	,675
Item 9: Carrying children	,681
Item 10: Taking care of an older adult	,679
Item 11: Sitting and using a computer or writing, while not at work	,686
Item 12: Watching TV or a video	,688
Item 13: Sitting and reading, talking, or on the phone, while not at work	,689
Item 14: Playing with pets	,689
Item 15: Light cleaning (make beds, laundry, iron, put things away)	,688
Item 16: Shopping (for food, clothes, or other items)	,688
Item 17: Heavier cleaning (vacuum, mop, sweep, wash windows)	,688
Item 19: Mowing lawn using a walking mower, raking, gardening	,689
Item 20: Walking slowly to go places (such as to the bus, work, visiting) (not for fun or exercise)	,686
Item 21: Walking quickly to go places (such as to the bus, work, or school) (not for fun or exercise)	,686
Item 22: Driving or riding in a car or bus	,684
Item 23: Walking slowly for fun or exercise	,688
Item 24: Walking more quickly for fun or exercise	,688
Item 25: Walking quickly up hills for fun or exercise	,688
Item 26: Jogging	,689
Item 27: Prenatal exercise class	,688
Item 28: Swimming	,689
Item 29: Dancing	,689
Item 32: Sitting at working or in class	,653
Item 33: Standing or slowly walking at work while carrying things (heavier than a 1-liter milk jug)	,659
Item 34: Standing or slowly walking at work not carrying anything	,648
Item 35: Walking quickly at work while carrying things (heavier than a 1-liter milk jug)	,703
Item 36: Walking quickly at work not carrying anything	,636
